# Prediction of lncRNA-disease association based on a Laplace normalized random walk with restart algorithm on heterogeneous networks

**DOI:** 10.1186/s12859-021-04538-1

**Published:** 2022-01-04

**Authors:** Liugen Wang, Min Shang, Qi Dai, Ping-an He

**Affiliations:** 1 School of Science, Zhejiang Sci-Tech University, Hangzhou, 310018 China; 2 College of Life Science, Zhejiang Sci-Tech University, Hangzhou, 310018 China

**Keywords:** lncRNA-disease associations, Similarity network, Heterogeneous network, LRWRHLDA, Ten-fold cross validation, AUC

## Abstract

**Background:**

More and more evidence showed that long non-coding RNAs (lncRNAs) play important roles in the development and progression of human sophisticated diseases. Therefore, predicting human lncRNA-disease associations is a challenging and urgently task in bioinformatics to research of human sophisticated diseases.

**Results:**

In the work, a global network-based computational framework called as LRWRHLDA were proposed which is a universal network-based method. Firstly, four isomorphic networks include lncRNA similarity network, disease similarity network, gene similarity network and miRNA similarity network were constructed. And then, six heterogeneous networks include known lncRNA-disease, lncRNA-gene, lncRNA-miRNA, disease-gene, disease-miRNA, and gene-miRNA associations network were applied to design a multi-layer network. Finally, the Laplace normalized random walk with restart algorithm in this global network is suggested to predict the relationship between lncRNAs and diseases.

**Conclusions:**

The ten-fold cross validation is used to evaluate the performance of LRWRHLDA. As a result, LRWRHLDA achieves an AUC of 0.98402, which is higher than other compared methods. Furthermore, LRWRHLDA can predict isolated disease-related lnRNA (isolated lnRNA related disease). The results for colorectal cancer, lung adenocarcinoma, stomach cancer and breast cancer have been verified by other researches. The case studies indicated that our method is effective.

**Supplementary Information:**

The online version contains supplementary material available at 10.1186/s12859-021-04538-1.

## Background

The disease is an abnormal life activity process that occurs due to the disorder of homeostasis after the body is damaged by the cause of the disease under certain conditions. Currently, many studies have confirmed that there is a complex cross-regulation relationship among diseases, genes, lncRNAs, and miRNAs [1–4].

Many researches have shown that although the proportion of encoded proteins in the human genome is less than 2%, under certain conditions, most of all nucleotides are detectably transcribed [5]. Among the various types of non-protein-coding transcripts, long non-coding RNAs (lncRNAs) and microRNAs (miRNAs) has attracted more and more attention. Among them, lncRNAs are defined as non-coding RNA with a length greater than 200 nucleotides [6]; miRNAs are an RNA molecule with a length of about 19–25 nucleotides that exists widely in eukaryotes [7].

The lncRNAs play an important role in a variety of biological mechanisms, such as epigenetic regulation, chromatin remodeling, gene transcription, protein transport, cell transportation [8]. The function of lncRNAs can be divided into the following categories: Transcription interference; Inducing chromatin remodeling and nucleosome modification; Regulating alternative splicing mode; Generating endogenous siRNAs; Regulating protein activity; Structure or Tissue function; Change the location of protein; Precursor of small RNA [5, 9, 10], et al*.*

Many researchers found that the expression or functional abnormalities of lncRNAs are closely related to the occurrence of human diseases, including cancers and degenerative neurological diseases, which seriously endanger human health. For example: The lncRNA HOTAIR overexpression increases breast cancer cell proliferation [11, 12]. The lncRNA AFAP1-AS1 has abnormal expression in cholangiocarcinoma, gallbladdercancer, hepatocellular carcinoma, gastric cancer, colorectal cancer, esophageal cancer [13]. The lncRNA HOXA-AS2 may be a biomarker for the treatment of gastric cancer, et al. [14]. There is a close correlation between lncRNA PCGEM1 and osteoarthritis [15]. Therefore, lncRNAs can be used as an important biomarker for the diagnosis of diseases.

The identification of lncRNA-diseases association includes biological experimental verification methods and computational model predictions. For example, based on the biological experiments, Faghihi et al. [16] found that the expression of BACE1-AS can promote the rapid feed forward regulation of β-secretase in Alzheimer’s disease. Applying the RT-PCR technology and Northern blot analysis, Hu et al. [17] confirmed and verified that H19 may become a new target for colon cancer anti-tumor therapy. The results of biological experimental are reliable, however, they are time-consuming and costly.

Recently, the computational model attracted more and more attention, in which various data resources can be integrated, to identify the lncRNA-disease association. For instance, based on a semi-supervised learning framework, the Laplacian regularized least squares for lncRNA-disease association calculation model (LRLSLDA) was suggested to predict potential disease-related lncRNA models [18]. Integrating genome, regulome and transcriptome data, the naive Bayesian classifier was proposed to identify cancer-related lncRNAs [19]. Similarly, based on disease-gene cluster association scores, a machine learning method was suggested to predict potential lncRNA-disease associations [20]. Combining the incremental principal component analysis (IPCA) and random forest (RF) algorithm, a machine learning model, called as IPCARF, was applied to predict the lncRNA-disease associations [21].

In the process of finding lncRNA-disease associations, the method of matrix factorization has also been widely used. For instance, the dual-network integrated logistic matrix factorization and Bayesian optimization model has been used for lncRNA-disease associations (DNILMF-LDA) [22]. In addition, the weighted graph regularized collaborative matrix factorization (WGRCMF), dual sparse collaborative matrix factorization (DSCMF) and the multi-label fusion collaborative matrix factorization (MLFCMF) were applied to construct model for prediction of lncRNA-disease associations [23–25].

Based on the hypothesis that lncRNAs with similar functions may be related to diseases with similar phenotypes, some researchers have proposed several calculation methods based on biological networks to predict disease-related lncRNAs.

In addition, integrating the lncRNA and the disease similarity network, and the lncRNA-disease association network. BPLLDA model based on paths of fixed lengths in a heterogeneous lncRNA-disease association network was proposed to predict lncRNA-disease associations [26]. Furthermore, some random walk models on these heterogeneous networks were suggested to predict the relationship between lncRNA and disease [27–29]. For example, Sun et al. [27] proposed the random walk with restart method on a lncRNA functional similarity network (RWRlncD). Gu et al. [28] proposed a global network-based random walk with restart algorithm on lncRNA seed nodes and disease seed nodes to predict the relationship between lncRNA and disease (GrWLDA). Based on the heterogeneous network through the lncRNA, disease, and gene similarity network, MHRWR model was proposed based on random walk with restart algorithm on the global network [29].

Following the random walk with restart model, in the paper, a new computational model based on Laplacian normalized random walk with restart algorithm in a heterogeneous network was proposed to predict the association between lncRNA and disease. Firstly, the disease semantic similarity (lncRNA function similarity, gene function similarity, miRNA function similarity) is calculated. And then, based on the association of lncRNA and disease (miRNA and gene), the Gaussian interaction profile kernel similarity of lncRNA and disease (miRNA and gene) are calculated. The lncRNA function similarity (disease semantic similarity, miRNA function similarity, gene function similarity) is integrated with the Gaussian interaction profile kernel similarity for lncRNAs (diseases, miRNAs, genes) to construct the isomorphic networks. Furthermore, the Laplace normalized random walk with restart algorithm on heterogeneous networks is developed to predict potential lncRNA-disease association. As a result, our method obtains reliable AUCs of 0.98402 in the ten-fold cross validation. The performance of our method is superior to other similar methods. Moreover, case studies on colorectal cancer, lung adenocarcinoma, stomach cancer and breast cancer also demonstrate the reliability of our model.

## Methods

### Experimental data sources

In the paper, the databases involved in lncRNA-disease associations mainly include LncRNADisease database [30, 31], EVLncRNAs database [32], Lnc2Cancer database [33], MNDR v3.1 database [34], et al. Similarly, the lncRNA-miRNA association comes from the integrated data of DIANA-LncBase database [35], LncAcTdb 2.0 database [36], MiRcode database [37], and StarBase database [38]. The lncRNA-gene association comes from the integrated data of LncRNADisease database [30, 31], LncAcTdb 2.0 database [36] and LncRNA2Target v2.0 database [39]. The miRNA-disease association comes from the integrated data of MNDR v3.1 database [34], HMDD database [40] and MiR2Disease database [41]. The miRNA-gene association comes from the data of MiRTarBase database [42]. The gene-disease association comes from the integrated data of DisGeNET database [43], CREEDS database [44], and DISEASES database [45].

Due to the different databases may have different names for the same biomolecule, so we need to perform data error correction and data cleaning on the data sets obtained from the database (mainly includes deleting duplicates, mistake, vacant data). In addition, the names of biomolecules of the same type from different databases are unified. In order to improve the comprehensiveness of the data and further improve the accuracy and scope of the prediction, the union of the related data of the above database was considered.

**Fig. 1:**
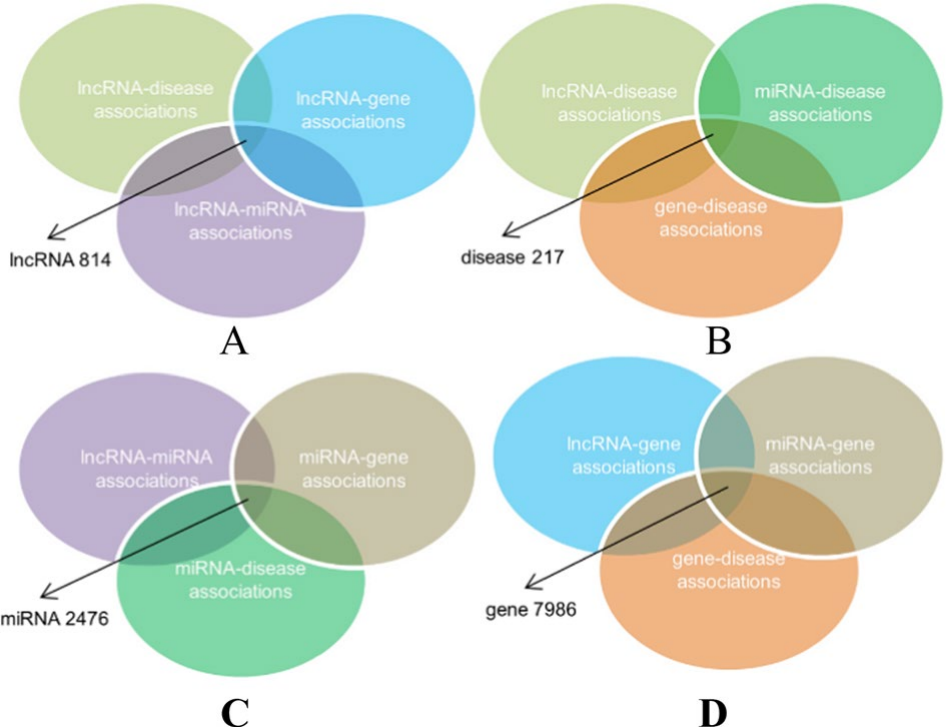
Ultimately retained the number of lncRNA, disease, miRNA, gene node. **A** lncRNA. **B** Disease. **C** miRNA. **D** Gene

For lncRNA, the intersection of three database, lncRNA-disease, lncRNA-gene and lncRNA-miRNA association set obtained from all databases, were considered to construct the lncRNA similarity network. There are 814 lncRNA in the work (Fig. 1). Finally, 2476 miRNAs, 7986 genes, and 217 diseases were remained to research. At the same time, we also summarize some basic characteristics of the X–Y association dataset (e.g., the average degree) of the dataset in Table 1. And X and Y both stand for lncRNA, disease, gene, miRNA.

**Table 1:** The basic characteristics of the X–Y association dataset

X	Y	Total	Total of associations	Average degree of X	Average degree of Y	Max degree of X	Max degree of Y
lncRNA		814					
	Disease	217	3434	4.2	15.8	90	418
	miRNA	2476	38,010	46.7	15.4	2284	98
	Gene	7986	14,987	18.4	1.9	2178	35
miRNA		2476					
	Disease	217	27,174	11.0	125.2	81	2453
	Gene	7986	216,934	87.6	27.2	1374	355
Gene		7986					
	Disease	217	37,277	4.7	171.8	74	6066

### Calculate the similarity matrix

#### LncRNA functional similarity matrix

Similar to the method of Sun et al. [27], the functional similarity of two lncRNAs was computed as following:

Supposing lncRNA *l*_1_ is associated with the disease group *D*_1_ ($D_{1} = \{ d_{1i} |1 \le i \le a\}$), and lncRNA *l*_2_ is associated with the disease group *D*_2_ ($D_{2} = \{ d_{2j} |1 \le j \le b\}$), the similarity between disease *d*_11_ and a disease group *D*_2_ is defined as follows:

1 $$S(d_{11} ,D_{2} ) = \mathop {\max }\limits_{{d_{2} \in D_{2} }} (Sim(d_{11} ,d_{2} )),$$

where $Sim(d_{11} ,d_{2} )$ is the disease semantic similarity of diseases *d*_11_ and *d*_2_. Then, the functional similarity between lncRNA *l*_1_ and *l*_2_ is defined as:

2 $$LS(l_{1} ,l_{2} ) = \frac{{\sum\limits_{1 \le i \le a} {S(d_{1i} ,D_{2} ) + \sum\limits_{1 \le j \le b} {S(d_{2j} ,D_{1} )} } }}{a + b}.$$

#### Disease semantic similarity matrix

The Disease Ontology (DO) provides open-source ontology for the integration of biomedical data that is associated with human disease [46]. The terms in DO are diseases or ideas of disease-related that are organized in a directed acyclic graph (*DAG*). Applying the method of Wang et al. [47, 48], the semantic similarity of diseases is calculated as following:

Given disease *d*, its *DAG* graph can be expressed as *DAG*(*d*) = (*Ans*(*d*), *E*(*d*)), where *Ans*(*d*) represents the set of the node, including node and its ancestor nodes, *E*(*d*) represents the edge set of the corresponding direct link from the parent node *d* to the child node. That is the *E*(*d*) denotes the relationship between different diseases. Based on *DAG* graph, the contribution of disease term *d* to the semantic value of disease *T* and the semantic value of disease *T* itself can be computed by the following two steps:

3 $$\left\{ \begin{gathered} D_{T} (d) = 1\quad \quad \quad \quad \quad \quad \quad \quad \quad \quad \quad \quad \quad \quad \;\;if\;d = T, \hfill \\ D_{T} (d) = \max \{ \Delta *D_{T} (d^{\prime})|d^{\prime} \in chidren\;of\;d\} \quad if\;d \ne T,\; \hfill \\ \end{gathered} \right.$$

4 $$DV(T) = \sum\limits_{d \in Ans(d)} {D_{T} (d)} ,$$

where $\Delta$ is the semantic contribution attenuation factor and its value ranged from 0 to 1. As the direct distance between disease *d* and its ancestor diseases increases, the contribution of these ancestral diseases to the semantic value of disease *d* will gradually decrease. The semantic similarity between diseased *d*_1_ and diseased *d*_2_ is calculated by Eq. (5):

5 $$DS(d_{1} ,d_{2} ) = \frac{{\sum\limits_{{d \in (Ans(d_{1} ) \cap Ans(d_{2} ))}} {(D_{{d_{1} }} (d) + D_{{d_{2} }} (d))} }}{{DV(d_{1} ) + DV(d_{2} )}}.$$

#### MiRNA functional similarity matrix

Similar to the Wang et al. [47] method, the functional similarity of two miRNAs can be defined as following:

Assuming that miRNA *m*_1_ is associated with the disease group *D*_3_ ($D_{3} = \{ d_{3k} |1 \le k \le c\}$) and miRNA *m*_2_ is associated with the disease group *D*_4_ ($D_{4} = \{ d_{4z} |1 \le z \le e\}$). The similarity of a disease *d*_31_ and a disease group *D*_4_ is defined as follows:

6 $$S(d_{31} ,D_{4} ) = \mathop {\max }\limits_{{d_{4} \in D_{4} }} (Sim(d_{31} ,d_{4} )),$$

and the functional similarity between miRNA *m*_1_ and *m*_2_ is computed by Eq. (7):

7 $$MS(m_{1} ,m_{2} ) = \frac{{\sum\limits_{1 \le k \le c} {S(d_{3k} ,D_{4} ) + \sum\limits_{1 \le z \le e} {S(d_{4z} ,D_{3} )} } }}{c + e}.$$

#### Gene function similarity matrix

The Gene Ontology (*GO*) database is the world’s largest informatics resource on the functions of genes [49]. For a *GO* node *A*, *DAG* = (*Ans*(*A*), *E* (*A*)) is its directed acyclic graph, where *Ans*(*A*) represents the set of all ancestors of node *A* (including node *A*); *E* (*A*) represents the set of edges connecting each node in *DAG*. For any *GO* node, assuming *t* is the ancestor of *A*, or *t* = *A*, $S_{A} (t)$ of *t*'s contribution to *A* is defined by Eq. (8):

8 $$\left\{ \begin{gathered} S_{A} (t) = 1\quad \quad \quad \quad \quad \quad \quad \quad \quad \quad \quad \quad \quad \;\;\;if\;t = A, \hfill \\ S_{A} (t) = \max \{ \Delta *S_{A} (t^{\prime})|t^{\prime} \in chidren\;of\;t\} \quad if\;t \ne A, \hfill \\ \end{gathered} \right.$$

where $\Delta$ is the semantic contribution attenuation factor and its value ranged from 0 to 1. As the direct distance between gene *A* and its ancestor genes increases, the contribution of these ancestral genes to the semantic value of gene *A* will gradually decrease. The semantic contribution $S_{V} (A)$ of node *A* is defined as follows:

9 $$S_{V} (A) = \sum {_{t \in Ans(A)} } S_{A} (t).$$

Then the semantic similarity of nodes *A* and *B* is calculated by Eq. (10):

10 $$S_{GO} (A,B) = \frac{{\begin{array}{*{20}c} {\sum {_{t \in (Ans(A) \cap t \in Ans(B))} } } & {(S_{A} (t) + S_{B} (t))} \\ \end{array} }}{{S_{V} (A) + S_{V} (B)}}.$$

The similarity of a go node *g* and a *GO* node set $G = \left\{ {{{\text{go}}_1},g{o_2}, \ldots ,g{o_f}} \right\}$ is defined as:

11 $$S(g,G) = \mathop {\max }\limits_{1 \le i \le f} (S_{GO} (g,go_{i} )).$$

Assuming that the GO term set annotations of genes *G*_1_ and *G*_2_ are $G{O_1} = \left\{ {{{\text{go}}_{11}},g{o_{12}}, \ldots ,g{o_{1m}}} \right\}$ and $G{O_2} = \left\{ {{{\text{go}}_{21}},g{o_{22}}, \ldots ,g{o_{2n}}} \right\}$, respectively, the similarity of the two genes *G*_1_ and *G*_2_ is calculated by Eq. (12) [50]:

12 $$GS(G_{1} ,G_{2} ) = \frac{{\sum\limits_{1 \le i \le m} {S(go_{1i} ,GO_{2} ) + \sum\limits_{1 \le j \le n} {S(go_{2j} ,GO_{1} )} } }}{m + n}.$$

### Gaussian interaction profile kernel similarity for lncRNAs and diseases

Because there are many zeros in the matrix *LS*, *DS*, *MS* and *GS*, this will cause the sparsity of the matrix, which may lead to the inaccuracy of the prediction results. To avoid such scenario, we introduce the Gaussian interaction profile kernel similarity [51, 52].

Firstly, the *m* × *n* matrix *LD* represents the association matrix of lncRNA and disease, the elements are only 0 and 1. For example, if lncRNA *l*_*i*_ is related to disease *d*_*j*_, *LD* (*i*, *j*) = 1, otherwise *LD* (*i*, *j*) = 0.

In the same way, we can define the lncRNA-miRNA association matrix *LM*, lncRNA-gene association matrix *LG*, disease-gene association matrix *DG*, miRNA-gene association matrix *MG*, miRNA-disease association matrix *MD*, respectively.

The Gaussian interaction profile kernel similarity of lncRNA *l*_*i*_ and *l*_*j*_ is defined as following:

13 $$GaL(l_{i} ,l_{j} ) = \exp ( - r_{l} ||IP(l_{i} ) - IP(l_{j} )||^{2} ),$$

14 $$r_{l} = r^{\prime}_{l} /(\frac{1}{m}\sum\limits_{i = 1}^{m} {||IP(l_{i} )} ||).$$

where *IP* (*l*_*i*_) is a binary vector, which represents the *i*th row of the lncRNA-disease association matrix *LD*, and *m* represents the number of lncRNAs. $r_{l}^{^{\prime}}$ is a regulation parameter of the kernel bandwidth parameter of $r_{l}$. According to the previous research, it is set to 1.

Similarly, the Gaussian interaction profile kernel similarity of disease *d*_*i*_ and *d*_*j*_ is defined as:

15 $$GaD_{{}} (d_{i} ,d_{j} ) = \exp ( - r_{d} ||IP(d_{i} ) - IP(d_{j} )||^{2} ),$$

16 $$r_{d} = r^{\prime}_{d} /(\frac{1}{n}\sum\limits_{i = 1}^{n} {||IP(d_{i} )} ||).$$

where *IP* (*d*_*i*_) is a binary vector, which represents the *i*th column of the lncRNA-disease association matrix *LD* and *n* is the number of diseases. $r^{\prime}_{d} = 1$, it is a regulation parameter of the kernel bandwidth parameter of $r_{d}$.

### Gaussian interaction profile kernel similarity for MiRNAs and genes

The Gaussian interaction profile kernel similarity calculation method of miRNA and gene is similar to that of lncRNA and disease, but the correlation matrix *MG* is used here. Therefore, we similarly define as follows: *IP* (*m*_*i*_)is a binary vector, which represents the *i*-th row of the matrix *MG* and *h* is the number of miRNAs. $r^{\prime}_{m}$ = 1, it is a regulation parameter of the kernel bandwidth parameter of $r_{m}$. *IP* (*g*_*i*_) is a binary vector, which represents the *i*th column of the matrix *MG* and *k* is the number of genes. $r^{\prime}_{g}$ = 1, it is a regulation parameter of the kernel bandwidth parameter of $r_{g}$.

### Integration of similarities between lncRNAs, miRNAs, genes, and diseases

We integrate the lncRNA functional similarity (disease semantic similarity, miRNA functional similarity, gene functional similarity) with the Gaussian interaction profile kernel similarity for lncRNAs (diseases, miRNAs, genes) as follows:

17 $$LL = \left\{ \begin{gathered} GaL(l_{i} ,l_{j} )\quad if\;l_{i} \;or\;l_{j} \in NL,\; \hfill \\ LS(l_{i} ,l_{j} )\quad \quad \quad \quad \quad \;\;\;\,else. \hfill \\ \end{gathered} \right.$$

18 $$DD = \left\{ \begin{gathered} GaD(d_{i} ,d_{j} )\quad \;if\;d_{i} \;or\;d_{j} \in ND,\quad \hfill \\ DS(d_{i} ,d_{j} )\quad \quad \quad \quad \quad \quad \;\;\,\,else. \hfill \\ \end{gathered} \right.$$

19 $$MM = \left\{ \begin{gathered} GaM(m_{i} ,m_{j} )\quad if\;m_{i} \;or\;m_{j} \in NM, \hfill \\ MS(m_{i} ,m_{j} )\quad \quad \quad \quad \quad \quad \quad \,else. \hfill \\ \end{gathered} \right.$$

20 $$GG = \left\{ \begin{gathered} GaG(g_{i} ,g_{j} )\quad if\;g_{i} \;or\;g_{j} \in NG, \hfill \\ GS(g_{i} ,g_{j} )\quad \quad \quad \quad \quad \quad \;\;\;else. \hfill \\ \end{gathered} \right.$$

where *NL* is the set of lncRNAs with no functional similarity with any other lncRNAs, *ND* is the set of diseases with no sematic similarity with any other disease, *NM* is the set of miRNAs with no functional similarity with any other miRNAs, and *NG* is the set of genes with no functional similarity with any other genes. By definition, *LL*, *DD*, *MM* and *GG* are symmetric.

### The heterogeneous network

**Fig. 2:**
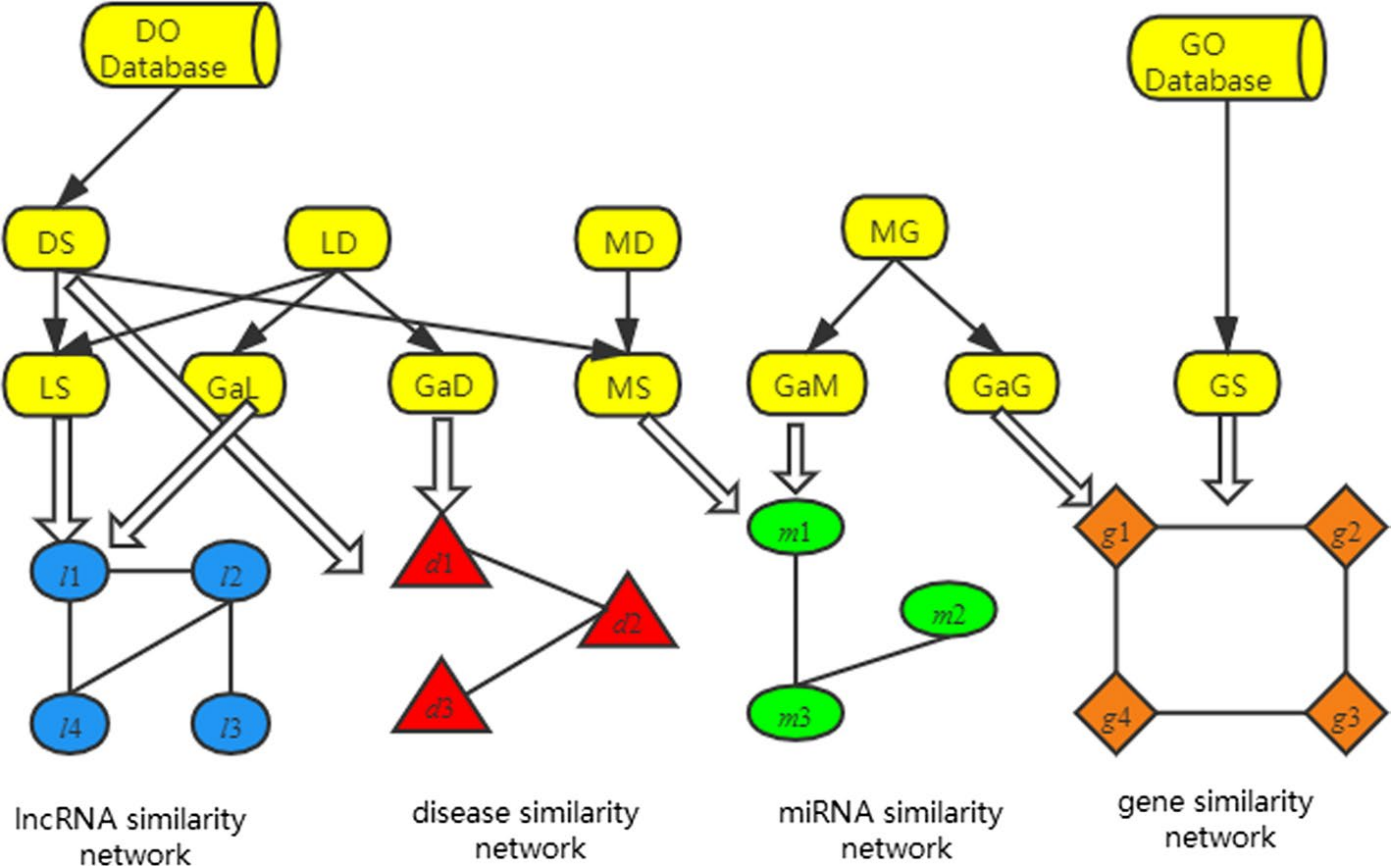
Construction of similarity network of lncRNAs, diseases, miRNAs and genes

**Fig. 3:**
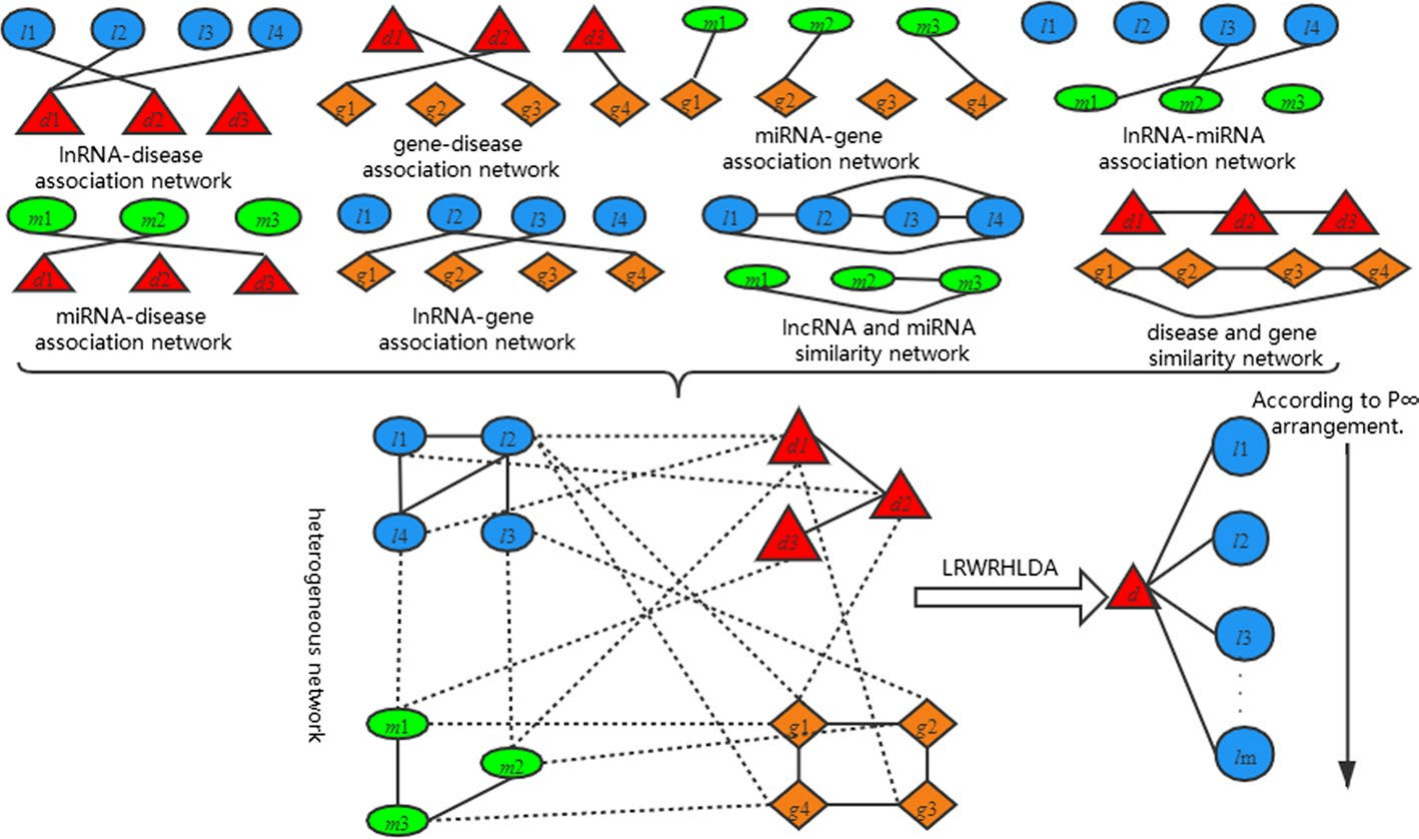
Construction of heterogeneous network, and rank of lncRNA according to the stable probability of lncRNA by LRWRHLDA

Based on the novel lncRNA similarity matrix *LL*, diseases similarity matrix *DD,* miRNA similarity matrix *MM*, and gene similarity matrix *GG*, four isomorphic networks include lncRNA similarity network, disease similarity network gene similarity network and miRNA similarity network were constructed, as shown in Fig. 2. In addition, a heterogeneous network through these four similarity networks and their interrelation ships were built based on six association matrix *LD, LM, LG, MD, MG, DG,* as shown in Fig. 3.

### The random walk with restart

Based on the heterogeneous network, the random walk with restart (RWR) on the heterogeneous network to predict lncRNA-disease association was defined as follows [53]:

21 $$P^{t + 1} = (1 - \lambda )WP^{t} + \lambda P^{0} ,$$

where *P*^0^ is the initial probability vector, *P*^*t*^ is the probability vector in which the *i*th element is the probability of detecting the random walk at node *i* at step *t*. λ is the restart probability, and its value ranged from 0 to 1. *W* is the probability transition matrix and *W*_*ij*_ denotes the transition probability from node *i* to *j*, when the *L*_1_ norm of *P*^*t*+1^ and *P*^*t*^ is less than 10^−6^, it can be considered that reaches a stable state, meanwhile, the stable probability $P^{\infty }$ can be obtained.

The probability transition matrix *W* is constructed in this paper as follows:

22 $$W = \left( {\begin{array}{*{20}c} {W_{LL} } & {W_{LM} } & {W_{LG} } & {W_{LD} } \\ {W_{ML} } & {W_{MM} } & {W_{MG} } & {W_{MD} } \\ {W_{GL} } & {W_{GM} } & {W_{GG} } & {W_{GD} } \\ {W_{DL} } & {W_{DM} } & {W_{DG} } & {W_{DD} } \\ \end{array} } \right).$$

Among them, the matrix *W* includes four intra-transition matrices and twelve inter-transition matrices. *W*_*LL*_ is the intra-transition matrix of lncRNA similarity network. *W*_*DD*_, *W*_*MM*_ and *W*_*GG*_ are similar to *W*_*LL*_ and represent the intra-transition matrix of disease similarity network, miRNA similarity network, and gene similarity network, respectively. *W*_*LM*_ is defined as the transition matrix from lncRNA network to miRNA network. *W*_*LG*_, *W*_*LD*_, *W*_*ML*_, *W*_*MG*_, *W*_*MD*_, *W*_*GL*_, *W*_*GM*_, *W*_*GD*_, *W*_*DL*_, *W*_*DM*_ and *W*_*DG*_ are defined similar to *W*_*LM*_.

### Laplacian normalization

Given the matrix *A* = *A* (*i*, *j*), the diagonal matrix *D* is defined as follows, if *i* = *j*, then *D* (*i*, *j*) is equal to the sum of the *i*th row of matrix *A*, otherwise *D* (*i*, *j*) = 0, then the Laplace normalization of matrix *A* is defined as [54, 55]:

23 $$\overrightarrow {A} (i,j) = \frac{A(i,j)}{{\sqrt {D(i,i)D(j,j)} }}.$$

Therefore, *W*_*LM*_ and *W*_*LL*_ can be obtained by the following two steps:

The probability of transition from *l*_*i*_ to *m*_*j*_ is as follows:

24 $$\overrightarrow {LM} (i,j) = \left\{ \begin{gathered} \frac{LM(i,j)}{{\sqrt {\sum\limits_{i} {LM} (i,j)\sum\limits_{j} {LM} (i,j)} }}\quad if\;\sum\limits_{i} {LM} (i,j)\sum\limits_{j} {LM} (i,j) \ne 0,\; \hfill \\ 0\quad \quad \quad \quad \quad \quad \quad \quad \quad \quad \quad \quad \quad \quad \quad \quad \quad \quad \quad \quad \quad else.\quad \hfill \\ \end{gathered} \right.$$

25 $$W_{LM} (i,j) = \left\{ \begin{gathered} P_{LM} *\frac{{\overrightarrow {LM} (i,j)}}{{\sum\limits_{j} {\overrightarrow {LM} (i,j)} }}\quad if\;\sum\limits_{j} {\mathop {LM}\limits^{ \to } (i,j)} \ne 0, \hfill \\ 0\quad \quad \quad \quad \quad \quad \quad \quad \quad \quad \quad \quad \quad \,else. \hfill \\ \end{gathered} \right.$$

The probability of transition from *l*_*i*_ to *l*_*j*_ is as follows:

26 $$\overrightarrow {LL} (i,j) = \left\{ \begin{gathered} \frac{LL(i,j)}{{\sqrt {\sum\limits_{i} {LL} (i,j)\sum\limits_{j} {LL} (i,j)} }}\quad if\;\sum\limits_{i} {LL} (i,j)\sum\limits_{j} {LL} (i,j) \ne 0, \hfill \\ 0\quad \quad \quad \quad \quad \quad \quad \quad \quad \quad \quad \quad \quad \quad \quad \quad \quad \quad \quad \;{\kern 1pt} {\kern 1pt} else. \hfill \\ \end{gathered} \right.\;$$

27 $$W_{LL} (i,j) = \left\{ {\begin{array}{*{20}l} {{{\overrightarrow {LL} (i,j)} \mathord{\left/ {\vphantom {{\overrightarrow {LL} (i,j)} {\sum\limits_{j} {\overrightarrow {LL} (i,j)} }}} \right. \kern-\nulldelimiterspace} {\sum\limits_{j} {\overrightarrow {LL} (i,j)} }}} \hfill & {if\;\sum\limits_{j} {\overrightarrow {LM} (i,j)} = 0,\sum\limits_{j} {\overrightarrow {LG} (i,j)} = 0,\sum\limits_{j} {\overrightarrow {LD} (i,j)} = 0,} \hfill \\ {(1 - P_{LM} )*{{\overrightarrow {LL} (i,j)} \mathord{\left/ {\vphantom {{\overrightarrow {LL} (i,j)} {\sum\limits_{j} {\overrightarrow {LL} (i,j)} }}} \right. \kern-\nulldelimiterspace} {\sum\limits_{j} {\overrightarrow {LL} (i,j)} }}} \hfill & {if\;\sum\limits_{j} {\overrightarrow {LM} (i,j)} \ne 0,\sum\limits_{j} {\overrightarrow {LG} (i,j)} = 0,\sum\limits_{j} {\overrightarrow {LD} (i,j)} = 0,} \hfill \\ {(1 - P_{LG} )*{{\overrightarrow {LL} (i,j)} \mathord{\left/ {\vphantom {{\overrightarrow {LL} (i,j)} {\sum\limits_{j} {\overrightarrow {LL} (i,j)} }}} \right. \kern-\nulldelimiterspace} {\sum\limits_{j} {\overrightarrow {LL} (i,j)} }}} \hfill & {if\;\sum\limits_{j} {\overrightarrow {LM} (i,j)} = 0,\sum\limits_{j} {\overrightarrow {LG} (i,j)} \ne 0,\sum\limits_{j} {\overrightarrow {LD} (i,j)} = 0,} \hfill \\ {(1 - P_{LD} )*{{\overrightarrow {LL} (i,j)} \mathord{\left/ {\vphantom {{\overrightarrow {LL} (i,j)} {\sum\limits_{j} {\overrightarrow {LL} (i,j)} }}} \right. \kern-\nulldelimiterspace} {\sum\limits_{j} {\overrightarrow {LL} (i,j)} }}} \hfill & {if\;\sum\limits_{j} {\overrightarrow {LM} (i,j)} = 0,\sum\limits_{j} {\overrightarrow {LG} (i,j)} = 0,\sum\limits_{j} {\overrightarrow {LD} (i,j)} \ne 0,} \hfill \\ {(1 - P_{LM} - P_{LG} )*{{\overrightarrow {LL} (i,j)} \mathord{\left/ {\vphantom {{\overrightarrow {LL} (i,j)} {\sum\limits_{j} {\overrightarrow {LL} (i,j)} }}} \right. \kern-\nulldelimiterspace} {\sum\limits_{j} {\overrightarrow {LL} (i,j)} }}} \hfill & {if\;\sum\limits_{j} {\overrightarrow {LM} (i,j)} \ne 0,\sum\limits_{j} {\overrightarrow {LG} (i,j)} \ne 0,\sum\limits_{j} {\overrightarrow {LD} (i,j)} = 0,} \hfill \\ {(1 - P_{LM} - P_{LD} )*{{\overrightarrow {LL} (i,j)} \mathord{\left/ {\vphantom {{\overrightarrow {LL} (i,j)} {\sum\limits_{j} {\overrightarrow {LL} (i,j)} }}} \right. \kern-\nulldelimiterspace} {\sum\limits_{j} {\overrightarrow {LL} (i,j)} }}} \hfill & {if\;\sum\limits_{j} {\overrightarrow {LM} (i,j)} \ne 0,\sum\limits_{j} {\overrightarrow {LG} (i,j)} = 0,\sum\limits_{j} {\overrightarrow {LD} (i,j)} \ne 0,} \hfill \\ {(1 - P_{LG} - P_{LD} )*{{\overrightarrow {LL} (i,j)} \mathord{\left/ {\vphantom {{\overrightarrow {LL} (i,j)} {\sum\limits_{j} {\overrightarrow {LL} (i,j)} }}} \right. \kern-\nulldelimiterspace} {\sum\limits_{j} {\overrightarrow {LL} (i,j)} }}} \hfill & {if\;\sum\limits_{j} {\overrightarrow {LM} (i,j)} = 0,\sum\limits_{j} {\overrightarrow {LG} (i,j)} \ne 0,\sum\limits_{j} {\overrightarrow {LD} (i,j)} \ne 0,} \hfill \\ {(1 - P_{LM} - P_{LG} - P_{LD} )*{{\overrightarrow {LL} (i,j)} \mathord{\left/ {\vphantom {{\overrightarrow {LL} (i,j)} {\sum\limits_{j} {\overrightarrow {LL} (i,j)} }}} \right. \kern-\nulldelimiterspace} {\sum\limits_{j} {\overrightarrow {LL} (i,j)} }}} \hfill & {if\;\sum\limits_{j} {\overrightarrow {LM} (i,j)} \ne 0,\sum\limits_{j} {\overrightarrow {LG} (i,j)} \ne 0,\sum\limits_{j} {\overrightarrow {LD} (i,j)} \ne 0.} \hfill \\ \end{array} } \right.$$

where *P*_*LM*_ (*P*_*LG*_, *P*_*LD*_) is the parameter which represents the transition probability from lncRNA similarity network to miRNA (gene, disease) similarity network and its value ranged from 0 to 1. Besides, *P*_*LM  *_= *P*_*ML*_, *P*_*LG  *_= *P*_*GL*_, *P*_*LD *_= *P*_*DL*_, *P*_*MG *_= *P*_*GM*_, *P*_*MD *_= *P*_*DM*_, *P*_*GD *_= *P*_*DG*_. Similarly, other intra-transition matrix and inter-transition matrix can be defined.Applying the Laplacian normalization, all elements of probability transition matrix *W* can be obtained.The calculation formula of *P*^0^ is as follows:

28 $$P^{0} = \left( {\begin{array}{*{20}c} {P_{L} *U_{L0} } \\ {P_{M} *U_{M0} } \\ {P_{G} *U_{G0} } \\ {(1 - P_{L} - P_{M} - P_{G} )*U_{D0} } \\ \end{array} } \right).$$

Among them, the parameters *P*_*L*_, *P*_*M*_, *P*_*G*_, 1 − *P*_*L *_− *P*_*M *_− *P*_*G*_ represent the importance of lncRNA similarity network, miRNA similarity network, gene similarity network and disease similarity network, respectively. Their values ranged from 0 to 1. *U*_*L*0_ represents the initial probability of the lncRNA similarity network, which is equal probabilities and is assigned to all seed nodes in the lncRNA similarity network. The sum of *U*_*L*0_ is 1. The initial probability *U*_*M*0_ and *U*_*G*0_ are similar to *U*_*L*0_. *U*_*D*0_ represents the initial probability of the disease similarity network, for disease *d*, the initial transition probability of disease *d* is 1, and the transition probability of other diseases is 0.

Finally, the Laplace normalized random walk with restart algorithm is used to predict related lncRNAs scores (see Fig. 3). The method was called as LRWRHLDA (the Laplace normalized random walk with restart algorithm in heterogeneous networks to predict the lncRNA-disease association).

## Results

### Performance evaluation

In this paper, ten-fold cross validation is used to evaluate the performance of our model. In the ten-fold cross validation, all known lncRNA-disease interactions are randomly divided into ten folds. For each experiment, nine subsets are regarded as training samples and the remaining one subset is treated as test samples. After completing the test, predicted scores are generated. Then, we rank test samples and unknown lncRNA-disease interactions. The corresponding predicted result of test samples is considered as true positive (TP) when the predicted relevance score is greater than the threshold. Otherwise, considered as false negative (FN). Similarly, for the unknown lncRNA-disease interactions, the corresponding predicted result consider as false positive (FP) when the predicted relevance score is greater than the threshold. Otherwise, considered as true negative (TN). Then, the true positive rates (TPR), the false positive rates (FPR), recall and precision are calculated as follow:

29 $$TPR = recall = \frac{TP}{{TP + FN}},$$

30 $$FPR = \frac{FP}{{FP + TN}},$$

31 $$precision = \frac{TP}{{TP + FP}}.$$

**Fig. 4:**
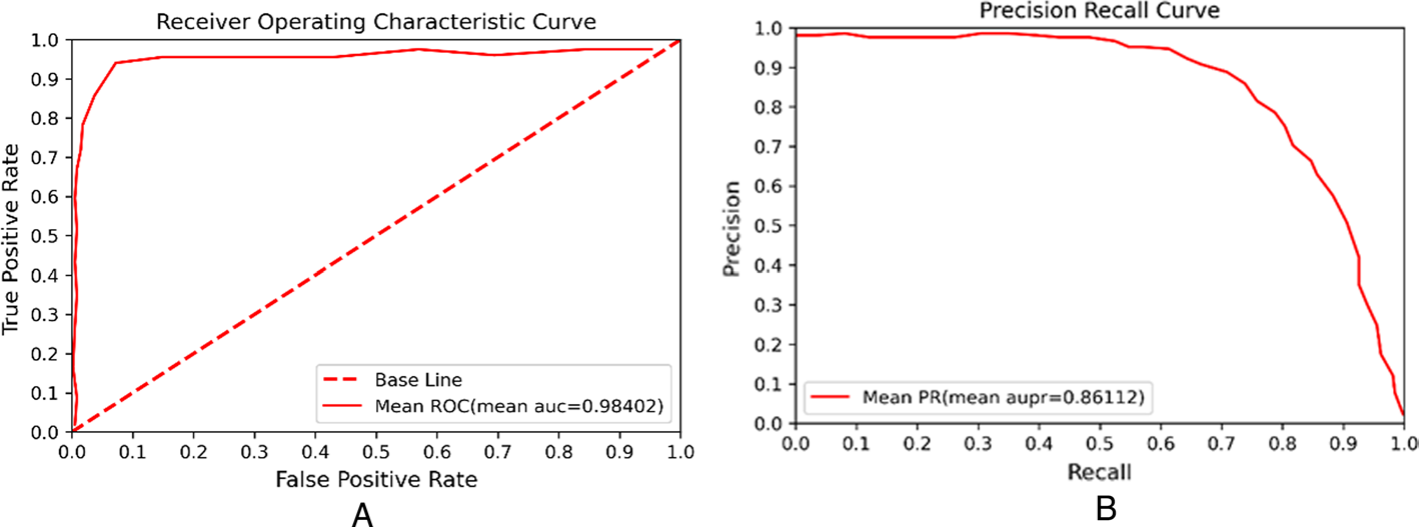
The performance of LRWRHLDA by ten-fold cross validation. **A** The average ROC curve. **B** The average PR curve

Finally, the receiver operating characteristic (ROC) curve and precision-recall curve (PR) curve are drawn as shown in Fig. 4. The area under the ROC curve (AUC) and the area under the PR curve (AUPR) are used to evaluate the performance of our method. The range of AUC, AUPR are all from 0 to 1. When the parameters are set to *P*_*LM*_ = *P*_*LG*_ = *P*_*LD*_ = *P*_*MG*_ = *P*_*MD*_ = *P*_*GD*_ = 0.2, *P*_*L*_ = 0.4, *P*_*M*_ = 0.1, *P*_*G*_ = 0.1, λ = 0.7, the results of ten experiments are shown in Table 2.

**Table 2:** The AUC (AUPR) value for each experiment and mean AUC (AUPR) value

Test	AUC	AUPR
Test 1	0.98284	0.85944
Test 2	0.98326	0.86120
Test 3	0.98416	0.86039
Test 4	0.98531	0.85861
Test 5	0.98435	0.86283
Test 6	0.98309	0.86184
Test 7	0.98497	0.86178
Test 8	0.98337	0.86041
Test 9	0.98374	0.86227
Test 10	0.98510	0.86240
Mean	0.98402	0.86112

### Comparison with different predicted methods using ten-fold cross validation

In order to compare with other models, the data in this paper is applied to the BPLLDA model [26], the RWRlncD model [27], GrwLDA model [28] and the MHRWR model [29].

**Fig. 5:**
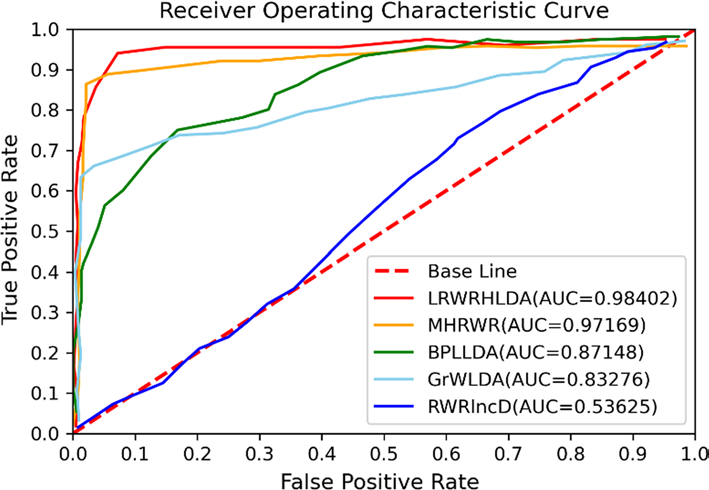
The ROC curve and AUC of LRWRHLDA, RWRlncD, GrWLDA, BPLLDA and MHRWR in predicting lncRNA-disease associations by the ten-fold cross validation

As a result, the ROC curves under ten-fold cross validation of LRWRHLDA, RWRlncD, GrwLDA, BPLLDA and MHRWR were plotted in Fig. 5.

As can be seen, LRWRHLDA has an AUC of 0.98402 and outperformed RWRlncD (0.53625), GrwLDA (0.83276), BPLLDA (0.87148) and MHRWR (0.97169). In summary, LRWRHLDA is better than other model in lncRNA-disease association prediction.

**Fig. 6:**
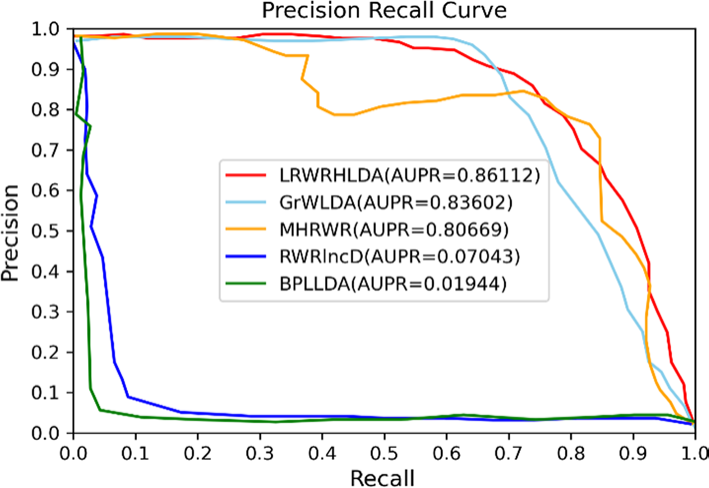
The PR curve and AUPR of LRWRHLDA, RWRlncD, GrWLDA, BPLLDA and MHRWR in predicting lncRNA-disease associations by ten-fold cross validation

The area under PR curve (AUPR) is also used to evaluate the performance of LRWRHLDA model, BPLLDA model [26], the RWRlncD model [27], GrwLDA model [28] and MHRWR model [29] to avoid overestimates the performance of these methods (see Fig. 6).

It can be seen from Fig. 6 that the AUPR value of LRWRHLDA is also higher than other models.

### Effects of parameters

There are ten parameters in our model, including the transition probability *P*_*LM*_, *P*_*LG*_, *P*_*LD*_, *P*_*MG*_, *P*_*MD*_, *P*_*GD*_ between networks; the weight of the subnet *P*_*L*_, *P*_*M*_, *P*_*G*_; and the restart probability λ. Due to too many parameters and our limited computing resources, we arbitrarily fixed nine of these parameters in the paper and only discussed the impact of restart probability λ with the ten-fold cross validation in our model. The results are shown in Table 3. As can be seen, based on the AUC index, the parameter λ has less influence on the performance of LRWRHLDA, when λ = 0.7. Based on the AUPR index, when λ is equal to 0.9, the AUPR value reaches the maximum. And observing Table 3, the results showed that the restart probability λ has powerful effects on our model.

**Table 3:** The AUC and AUPR values when λ taking different values from 0.1 to 0.9, in which other parameters were fixed

λ	AUC	AUPR
0.1	0.95693	0.45169
0.2	0.96973	0.57582
0.3	0.97582	0.66525
0.4	0.97947	0.73139
0.5	0.98184	0.78217
0.6	0.98338	0.82477
0.7	0.98402	0.86112
0.8	0.98346	0.89130
0.9	0.98058	0.91417

*P*_*LM*_*, P*_*LG*_*, P*_*LD*_*, P*_*MG*_*, P*_*MD*_*, P*_*GD*_ are all 0.2, *P*_*M*_ = 0.1, *P*_*G*_ = 0.1 and *P*_*L*_ = 0.4

## Case study

### Case studies on predicted lncRNA-disease associations

It is known that lncRNAs play critical roles in the development of many diseases. To evaluate the ability of LRWRHLDA in inferring potential lncRNA-disease associations, we use all known lncRNA-disease associations in LD as training data to assess the potential of predicted associations by our model.

The stable probability $P^{\infty }$ can be used as a measure of proximity to the seed lncRNAs. If $P^{\infty }$ (lncRNA *i*) > $P^{\infty }$ (lncRNA *j*), then lncRNA *i* will be in closer proximity to the seed lncRNAs than lncRNA *j* in the lncRNA similarity network. As a result, all candidate lncRNAs can be ranked according to the $P^{\infty }$, and the top ranked lncRNAs can be expected to have a high probability of being associated with the disease of interest. The novel lncRNA-disease associations are ranked according to the stable probability of LRWRHLDA. To validate the predictions, we use literature or the following those databases: LncRNADisease [30], LncRNADisease v2.0 [31], MNDR v3.1 [34], lnCAR [56]. Specifically, we list the top 10 lncRNAs associated with four diseases, including colorectal cancer, lung adenocarcinoma, stomach cancer and breast cancer. According to $P^{\infty }$, the top 10 results were shown in Table 4 (the detailed results see Additional file 1: Table-S1).

**Table 4:** The predicted top10 potential lncRNAs for four cancers by LRWRHLDA

Rank	LncRNA	Evidence	LncRNA	Evidence
Colorectal cancer			Lung adenocarcinoma	
1	CASC19	MNDR v3.1	ZNF295-AS1	MNDR v3.1
2	ENST00000535511	PMID: 28177879	LINC01969	MNDR v3.1
3	RP4	PMID: 29531464	PRKCZ-AS1	MNDR v3.1
4	CTNNAP1	PMID: 27487124	PIK3CD-AS2	MNDR v3.1
5	LINC01021	PMID: 29262524	GMDS-AS1	PMID: 31860169
6	UCOO2KMD.1	MNDR v3.1	FAM83A-AS1	MNDR v3.1
7	UICLM	MNDR v3.1	ACTA2-AS1	MNDR v3.1
8	UCC	MNDR v3.1	LINC00635	MNDR v3.1
9	N-BLR	MNDR v3.1	LINC01207	PMID: 26693067
10	RP11-317J10.2	MNDR v3.1	LINC00941	MNDR v3.1
Stomach cancer			Breast cancer	
1	M59227	MNDR v3.1	LNC015192	MNDR v3.1
2	LOC150622	MNDR v3.1	LINC00993	MNDR v3.1
3	MUC2	MNDR v3.1	LINC00894-002	MNDR v3.1
4	AKR7L	MNDR v3.1	AC008268.1	MNDR v3.1
5	AC110615.1	MNDR v3.1	MIR2052HG	MNDR v3.1
6	AC079089.1	MNDR v3.1	ST8SIA6-AS1	MNDR v3.1
7	PWRN1	MNDR v3.1	PAX8-AS1-N	MNDR v3.1
8	SUCLG2-AS1	MNDR v3.1	PRLB	MNDR v3.1
9	AL162586.1	MNDR v3.1	PDCD4-AS1	MNDR v3.1
10	LINC01856	MNDR v3.1	ADARB2-AS1	MNDR v3.1

Colorectal cancer is the third most common cancer diagnosed in the US. While the incidence and the mortality rate of colorectal cancer has decreased due to effective cancer screening measures, there has been an increase in number of young patients diagnosed in colon cancer due to unclear reasons at this point of time [57]. Lung adenocarcinoma is one of the main types of lung cancer, which belongs to non-small cell carcinoma. The incidence of lung adenocarcinoma is mainly female and non-smokers [58]. Stomach cancer is the fifth most common cancer and the third most common cause of cancer death globally [59]. The most majority of stomach cancers are adenocarcinomas, with no obvious symptoms in the early stage. They are often similar to the symptoms of chronic gastric diseases such as gastritis and gastric ulcers, and easily ignore. Moreover, the current early diagnosis rate of stomach cancer is still low. Breast cancer is a malignant tumor that occurs in the epithelial tissue of the breast. At present, breast cancer has become a major public health problem in the current society, and its cause is not yet fully understood. In the world, breast cancer is an important cause of human suffering and premature mortality among women [60].

In Table 4, the six potential lncRNA-disease associations were confirmed in the literature except the existing lncRNA-disease associations in the database, in which included ENST00000535511-colorectal cancer, RP4-colorectal cancer, CTNNAP1-colorectal cancer, LINC01021-colorectal cancer, GMDS-AS1-lung adenocarcinoma, LINC01207-lung adenocarcinoma. These results demonstrated that the predictive performance of the proposed method.

### Case studies on predicted novel diseases and novel lncRNAs

For each disease, it is deemed as a novel disease and all its related lncRNAs are removed to predict potential lncRNAs related the disease. All the candidate lncRNAs were ranked according to $P^{\infty }$ and lncRNAs with high scores were expected to be potentially related with investigated disease *d*. Depend on $P^{\infty }$, the top 10 results were listed in Table 5 (the detailed results see Additional file 2: Table-S2).

**Table 5:** The predicted top 10 novel lncRNAs-related for four cancers by LRWRHLDA

Rank	LncRNA	Evidence	LncRNA	Evidence
Colorectal cancer			Lung adenocarcinoma	
1	CARL	Unconfirmed	FOXP4-AS1	lnCAR
2	CASC19	MNDR v3.1	NEXN-AS1	lnCAR
3	MCM3AP-AS1	PMID: 32982409	VPS9D1-AS1	lnCAR
4	AL358334.2	lnCAR	TERC	lnCAR
5	AL157400.4	lnCAR	AC018413.1	unconfirmed
6	LAMA5-AS1	lnCAR	AL157838.1	lnCAR
7	HNF1A-AS1	MNDR v3.1	TUBB2A	unconfirmed
8	RGMB-AS1	lnCAR	AC019197.1	lnCAR
9	C21ORF62-AS1	lnCAR	Z93930.2	lnCAR
10	CASC8	MNDR v3.1	SATB2-AS1	PMID: 34249715
Stomach cancer			Breast cancer	
1	SSBP3-AS1	lnCAR	LINC00652	lnCAR
2	AC103740.1	lnCAR	TAPT1-AS1	lnCAR
3	AF117829.1	Unconfirmed	AC007823.1	lnCAR
4	AC092910.3	lnCAR	LHX1-DT	PMID: 33194577
5	RAB30-AS1	lnCAR	KLF3-AS1	MNDR v3.1
6	AC093157.1	lnCAR	FGF14-AS2	MNDR v3.1
7	GATA2-AS1	lnCAR	KCNK15-AS1	MNDR v3.1
8	PCA3	lnCAR	AC107959.2	lncRNADisease v2.0 (predicted)
9	TERC	MNDR v3.1	AP003486.1	Unconfirmed
10	AC087164.1	lnCAR	LINC00993	MNDR v3.1

Analogously, the stable probability $P^{\infty }$ can be also used as a measure of proximity to the seed diseases. All the candidate diseases were ranked according to $P^{\infty }$ and diseases with high scores were expected to be potentially related with investigated lncRNA. To evaluate the ability of our model to predict new lncRNAs, we analyzed two lncRNAs including H19 and HOTAIR. For each lncRNA, it is removed all its related diseases in predicting potential diseases. According to $P^{\infty }$, the top 10 results were showed in Table 6 (the detailed results see Additional file 3: Table-S3).

**Table 6:** The predicted top 10 novel diseases-related for H19 and HOTAIR by LRWRHLDA

H19			HOTAIR	
Rank	Disease	Evidence	Disease	Evidence
1	Carcinoma	lncRNADisease v2.0	Parkinson's disease	MNDR v3.1
2	Parkinson's disease	MNDR v3.1	Carcinoma	lncRNADisease v2.0
3	Colon cancer	MNDR v3.1	Colon cancer	MNDR v3.1
4	Stomach cancer	MNDR v3.1	Liver cancer	MNDR v3.1
5	Liver cancer	MNDR v3.1	Stomach cancer	MNDR v3.1
6	Pancreatic cancer	MNDR v3.1	Pancreatic cancer	MNDR v3.1
7	Kidney cancer	MNDR v3.1	Colorectal cancer	MNDR v3.1
8	Schizophrenia	lncRNADisease v2.0 (predicted)	Kidney cancer	MNDR v3.1
9	Colorectal cancer	MNDR v3.1	Colorectal carcinoma	MNDR v3.1
10	Glioblastoma	lncRNADisease	Melanoma	lncRNADisease

Observing Table 5, we can find that thirty-five of the top ten lncRNAs associations with four cancers were validated by the database or literature. However, other five cancer-lncRNA associations, colorectal cancer-CARL, stomach cancer-AF117829.1, breast cancer-AP003486.1, lung adenocarcinoma-AC018413.1 and lung adenocarcinoma-TUBB2A have not been confirmed by the database or literature. It implies our method can predict more additional lncRNA-disease associations.

From Table 6, in both cases, all top ten associated diseases were validated by the database. In summary, LRWRHLDA achieves favorable performances in predicting novel disease-associated lncRNAs and novel lncRNA-associated diseases.

## Discussion

At present, many studies have shown that lncRNA has an important influence on the physiological process of diseases. Because traditional biological experiments are time-consuming and costly, it is necessary to develop a computational model to predict the association between lncRNA and disease.

In this paper, a new model-LRWRHLDA based on the Laplace normalized random walk with restart algorithm in heterogeneous network was constructed to predict potential lncRNA-disease associations. The ten-fold cross validation test is applied to evaluate the prediction performance of our method. In comparison with the state-of-the-art prediction methods, our method can achieve better performance in terms of AUC values. Moreover, case studies of colorectal cancer, lung adenocarcinoma, stomach cancer and breast cancer are implemented to further demonstrate that it could be a useful method for predicting potential relationships between lncRNAs and diseases as well.

However, our method has some limitations. Firstly, since we have 10 parameters, the selection and adjustment of parameters still face some difficulties. Secondly, because of our model is based on four networks, there are too many nodes in the network. In the random walk process, the more nodes there are, the longer the random walk time will be. In the future, we will continue to improve the model.

## Conclusion

In this study, we proposed an effective method, LRWRHLDA, which is based on the Laplace normalized random walk with restart algorithm in heterogeneous network to predict the potential lncRNA and disease association. First, a heterogeneous network based on lncRNA, disease, miRNA, gene similarity network and their correlation networks were constructed. Then, we calculate the probability transition matrix by Laplace normalization. Finally, the potential lncRNA-disease associations were predicted by the random walk with restart over heterogeneous networks. Furthermore, LRWRHLDA can predict isolated disease-related lnRNA (isolated lnRNA-related disease). Our method is evaluated comprehensively by ten-fold cross validation and case studies in comparison with other methods. The results show that our method has higher prediction accuracy.

## Supplementary Information

**Additional file 1**. In this file we provide the results of stable probability of lncRNA when LRWRHLDA run over for four cancers based on the LD matrix.

**Additional file 2**. In this file we provide the results of stable probability of lncRNA when LRWRHLDA run over when delete related lncRNAs of the cancer.

**Additional file 3**. In this file we provide the results of stable probability of lncRNA when LRWRHLDA run over when delete related cancer of the lncRNAs.

## Data Availability

The datasets supporting the conclusions of this article are included within the article and its additional files. The code (executable code and source code) and data for this study are available at https://github.com/wang-124/LRWRHLDA.git.

## Abbreviations

lncRNAs: Long non-coding RNAs
miRNA: MicroRNA
LRWRHLDA: Prediction the potential lncRNA-disease associations based on Laplace normalized random walk with restart algorithm in heterogeneous networks
ROC: Receiver operating characteristic
TPR: True positive rates
FPR: False positive rates
AUC: Areas under ROC curve
PR: Precision-recall
AUPR: The area under the precision-recall curve
